# A Personalized Diagnosis Method to Detect Faults in a Bearing Based on Acceleration Sensors and an FEM Simulation Driving Support Vector Machine

**DOI:** 10.3390/s20020420

**Published:** 2020-01-11

**Authors:** Xiaoyang Liu, Haizhou Huang, Jiawei Xiang

**Affiliations:** 1 College of Mechanical and Electrical Engineering, Wenzhou University, Wenzhou 325035, China; liuxy199415@163.com; 2 Huadian Electric Power Research Institute, Hangzhou 310030, China; haizhou-huang@chder.com

**Keywords:** personalized fault diagnosis, bearings, finite element method, numerical simulation, support vector machines

## Abstract

Classification of faults in mechanical components using machine learning is a hot topic in the field of science and engineering. Generally, every real-world running mechanical system exhibits personalized vibration behaviors that can be measured with acceleration sensors. However, faulty samples of such systems are difficult to obtain. Therefore, machine learning methods, such as support vector machine (SVM), neural network (NNs), etc., fail to obtain agreeable fault detection results through smart sensors. A personalized diagnosis fault method is proposed to activate the smart sensor networks using finite element method (FEM) simulations. The method includes three steps. Firstly, the cosine similarity updated FEM models with faults are constructed to obtain simulation signals (fault samples). Secondly, every simulation signal is separated into sub-signals to solve the time-domain indexes to generate the faulty training samples. Finally, the measured signals of unknown samples (testing samples) are inserted into the trained SVM to classify faults. The personalized diagnosis method is applied to detect bearing faults of a public bearing dataset. The classification accuracy ratios of six types of faults are 90% and 92.5%, 87.5% and 87.5%, 85%, and 82.5%, respectively. It confirms that the present personalized diagnosis method is effectiveness to detect faults in the absence of fault samples.

## 1. Introduction

With the rapid development of artificial intelligence (AI) technology, machine learning methods have been widely used in fault diagnosis of mechanical components. Rolling bearings, as important components in rotating machinery, they exhibit personalized vibration behaviors under different work conditions. The running state of bearings directly affects the reliability and stability of the entire machine. Thus, in order to detect the faults in bearings, many researchers have proposed different intelligent diagnosis methods based on machine learning methods or artificial intelligence models [1,2,3]. Wang et al. [4] presented convolutional neural network-based hidden Markov models (CNN-HMMs) to classify multi faults in a bearing. To enlarge the application cases of training samples collected from fault simulators, an intelligent fault diagnosis approach was proposed using transfer learning to transfer fault samples from laboratory bearings to locomotive bearings [5]. By using limited fault samples as training and testing samples, the performance of machine learning methods have been verified to detect faults in bearings, such as extreme learning machine (ELM), support vector machine (SVM), neural networks (NNs) [6,7,8,9], etc. However, in the real world, it is difficult to obtain sufficient suitable training samples to represent various kinds of bearing faults that may occur in actual mechanical systems. Therefore, various machine learning methods have been greatly limited in engineering applications for the lack of fault training samples.

To fully understand the fault mechanism and obtain agreeable fault detection results, many researchers performed numerical simulations to analyze faults. To detect faults online in nonlinear continuous systems, Bregon et al. [10] used the simulation and state observer models to obtain the final fault diagnosis results. To detect the abnormal conditions of structures, two kinds of wavelet-based numerical simulation models were carried out to calculate the dynamic responses of a shaft [11,12]. Thereafter, Xiang and Zhong [13] further proposed a novel fault diagnosis scheme using finite element method (FEM), wavelet packet transform (WPT), and SVM. To detect faults in reciprocating machine, Wang et al. [14] proposed a combination method using the minimum entropy deconvolution (MED) and FEM modal analysis determined band-pass filter to detect faults in an axial piston pump. Generally, as a powerful tool, FEM simulation can be employed to obtain a large number of numerical data/signals at a lower experimental cost, especially for those that are difficult to obtain through physical experiments. 

FEM models are constructed on the basis of a highly idealized engineering design, and the dynamic responses of FEM simulations and physical experiments are usually quite different. In order to obtain effective FEM simulation results similar to those in real-world running mechanical systems, it is necessary to update the FEM model. As an optimization problem, the FEM model updating is achieved by correcting the design parameters, such as boundary conditions and materials [15]. According to the dynamic theory of mechanical system, the dynamic information in different assembly and working states will be carried in the corresponding vibration signals [16]. Therefore, various FEM model parameters can be corrected effectively by comparing the measured signals with the FEM simulation signals using similarity indexes, such as the cosine similarity, Theil’s inequality coefficient, etc. [17]. 

As one of the key problems in the intelligent diagnosis, the selection of feature vectors is affect diagnosis result directly. Generally, feature vectors are generated by calculating the feature indexes in time, frequency and time-frequency domains. Chen et al. [18] proposed an approach including six indexes (e.g., mean value, root mean square, standard deviation, skewness, kurtosis and shape indicator) and two indexes (e.g., mean frequency and standard deviation frequency) in the time frequency domains, respectively to construct a feature set to train the SVM. Liu et al. [19] used a hybrid time frequency analysis method to get the feature information to classify gear faults.

SVM is a pattern recognition approach widely used in fault classification [20]. Based on Vapnik Chervonenkis (VC) dimension theory and structural risk minimization (SRM) principle, SVM can be used to solve the nonlinear and high dimensional problems. However, due to the lack of fault training samples, SVM has not been successfully applied to real-world running mechanical systems.

Recently, motivated by personalized medicine, Xiang [13] developed a personalized diagnosis concept for mechanical fault diagnosis by the combination of numerical simulations and machine learning methods or artificial intelligence models. If the FEM model can effectively represent the real-world running mechanical systems, the lack of fault training samples will be inexpensively obtained by FEM simulations. Thereafter, machine learning methods or artificial intelligence models will be activated for the diagnosis of mechanical faults under different working conditions. In this paper, we proposed a new personalized diagnosis method called FEM simulation driving SVM to detect faults in a bearing. In Section 2, the basic principle of the personalized diagnosis method is introduced. In Section 3, an example is given to show the performance of the present method to classify six types of faults in experimental test rigs. Concluding remarks of this study are given in Section 4.

## 2. The Basic Principle of Personalized Diagnosis Method

### 2.1. Cosine Similarity-Based FEM Model Updating Technique

As we know, vibration of mechanical system is the external reflection of its intrinsic dynamic characteristics, thus vibration signals (dynamic responses) can be used to determine the corresponding main parameters of FEM model, such as materials and boundary conditions. Many researchers have done researches on model parameter identification and fault diagnosis based on vibration signals [21,22,23]. Sarin et al. [24] made comparative study residual errors between time-domain signals of simulations and experiments, and further provided a theoretical basis for model updating. Zapico-Valle et al. [25] defined a residual value between FEM simulation and experimental signals to search the minimum residual value using optimization methods for the purpose of FEM model updating. Here, we directly use the cosine similarity between the time-domain vibration signals of FEM simulation and experimental measurement to update the FEM model of a bearing by adjusting the corresponding parameters. The cosine similarity between the measured and the FEM simulation signals is defined by:

(1) $$cos\left(\theta \right)=\sum_{i=1}^{n}\left(x_{i}\times y_{i}\right)/\left(\sqrt{\sum_{i=1}^{n}{\left(x_{i}\right)}^{2}}\times \sqrt{\sum_{i=1}^{n}{\left(y_{i}\right)}^{2}}\right)=\frac{X\cdot Y}{\left\|X\right\|\times \left\|Y\right\|},$$

where *X* and *Y* represent the measured and simulation signals, respectively. When the closer the value of *cos*(*θ*) is to 1, the more similar the two signals are. Generally, in engineering applications, *cos*(*θ*) > 0.6 will lead to a satisfactory result [26].

### 2.2. A Brief Review of SVM

Consider a training set S:

(2) $$S={\left\{x_{i},y_{i}\right\}}_{i=1^{\prime }}^{l},$$

where *x_i_* Є R*^l^*, *y_i_* Є {-1,+1}, and *l* is the number of samples.

The aim of SVM is to determine an optimal hyper plane for separating one from the others by using the training dataset. To get the ideal hyper plane, the dual optimization problem is often mentioned in SVM as:

(3) $$min-\frac{1}{2}\sum_{i=1}^{l}\sum_{j=1}^{l}\alpha_{i}\alpha_{j}y_{i}y_{j}K\left(x_{i},y_{j}\right)+\sum_{i=1}^{l}\alpha_{i},s.t.\sum_{i=1}^{l}y_{i}\alpha_{i}\begin{array}{cc} & 0\leq \alpha_{i}\leq C,i=1,2,\ldots ,l \end{array},$$

where *α_i_* is the Lagrange multiplier coefficient obtained by dealing with the dual optimization in the process of the SVM training; *K(x_i_, y_i_)* is referred to as the kernel function; *C* > 0 is the error penalty parameter. 

There are many forms of the kernel function. The radial basis function (RBF) kernel is employed in this paper for the highest accuracy ratio of classification. How to choose the tradeoff parameters *ρ*_1_ (the width of RBF) and *C* is a difficult task in the application of SVM. A possible way is suggested by Xiang et al. [27] suggested that the simulation investigation should proceed first to obtain the relative best parameters *ρ*_1_ and *C*, and hence, *ρ*_1_ = 1.05 and *C* = 10 were employed in the present investigation.

### 2.3. The Personalized Diagnosis Method by Using FEM Simulation and SVM

**Figure 1:**
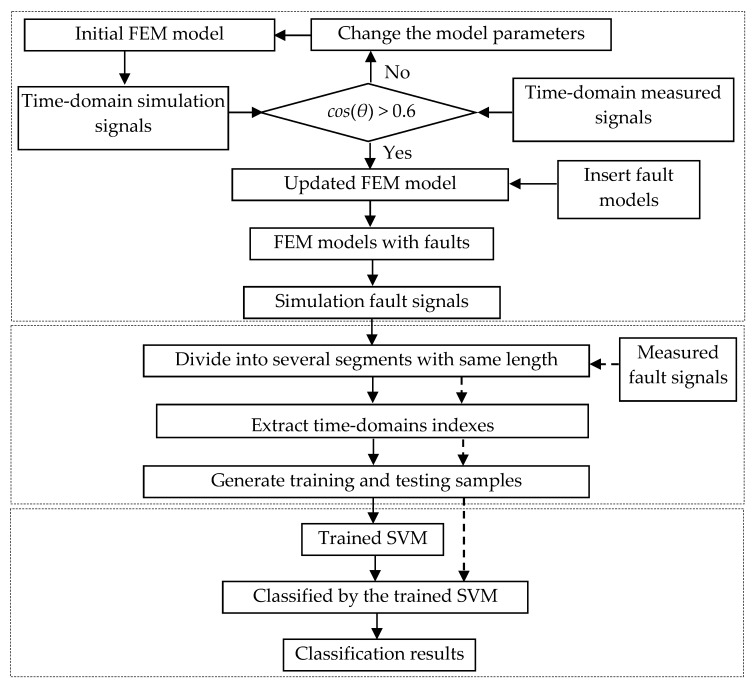
The flowchart of the personalized fault diagnosis method.

For the bearings under different running sates, the vibration response signals exhibit personalized characteristics. In the fault diagnosis of bearings, a generalized fault samples definitely not available for all the bearings. Therefore, a new idea for personalized fault diagnosis is developed using FEM simulation to activate SVM. Figure 1 shows the flowchart of the present method.

#### 2.3.1. Construct and Update the FEM Model for a Mechanical System

FEM model of a mechanical systems is constructed using the commercial FEM software ANSYS. To keep the FEM model effectively represent the corresponding physical mechanical system, the cosine similarity-based FEM model updating technique is used to determine the model parameters based on the comparison of the time-domain signals obtained by FEM simulations and measurements. If the value of cosine similarity is larger than 0.6, the FEM model can well represent its physical one. Moreover, the faulty models will be inserted into the FEM model to simulate the dynamic response of mechanical system with faults.

#### 2.3.2. Obtain the Faulty Training Samples

The FEM models of the mechanical systems with different faults are calculated to obtain the simulation vibration signals in time-domain. Then, each simulation signal is divided into sub-signals with the same length in time domain, and the sixteen time-domain indexes (as shown in Table 1) of each sub-signal are further calculated. Therefore, the number of training samples corresponding to each fault is the same, which ensures the balance of the samples.

#### 2.3.3. Fault Classification Using SVM

The fault training samples formed by the time-domain indexes are used as inputs to train SVM. Same as the processing of simulation signals, each measured signal (the fault type is unknown) is employed to generate the testing samples to the trained SVM, and fault patterns are finally identified. 

## 3. Experimental Investigations

In this section, experimental investigations of a public bearing dataset were carried out, which proves that the personalized diagnosis method is feasible in bearing faults diagnosis using FEM simulation driving SVM. In order to ensure the reliability of the measured signals, the bearing data are from the Bearing Data Centre at Case Western Reserve University (CWRU), as referred to in the website [28]. The drive-end bearing is taken as the experimental object, and the sampling frequency is 6 kHz.

### 3.1. Construction and Updating of Bearing FEM Model 

**Figure 2:**
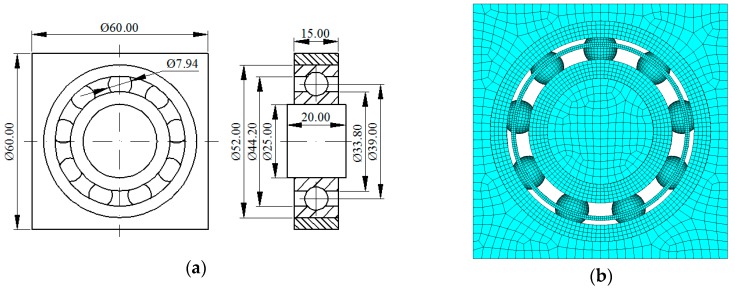
The geometrical dimension and finite element method (FEM) model of a bearing: (**a**) the geometrical dimension and (**b**) the FEM model.

According to the experiment of CWRU, the geometrical dimensions of bearing were determined, shown in Figure 2a. Using commercial finite element analysis (FEA) software ANSYS to construct a three-dimensional (3D) FEM model of the bearing with the bearing seat and shaft, as shown in Figure 2b.

In constructing the FEM model of the bearing, 3D solid element (SOLID164) and shell element (SHELL163) are employed to mesh the body and inner surface of inner ring (for applying the rotating loading), respectively. In order to reduce the computing time and improve the calculation accuracy, the element size is changed according to the components of bearing, as shown in Table 2. Figure 2b shows the result of meshing, and the total FEM model contains 71,976 elements with 73,133 nodes. All the components of bearing are constructed with line elastic material: density ρ = 7860 kg/m^3^, elastic modulus E = 2.06 × 10 Pa, and Poisson’s ratio μ = 0.3.

Consider the actual working condition, three contact pairs are defined in the FEM model:

(1) *S*_1_, a contact pair between the balls and inner ring.

(2) *S*_2_, a contact pair between the balls and outer ring.

(3) *S*_3_, a contact pair between the outer ring and bearing seat.

Due to the max radial load of bearing is known to be *F_r_* = 14 kN, the dynamic friction coefficients *f*_1_ and *f*_2_ of *S*_1_ and *S*_2,_ respectively, can be determined using the geometry and material parameters [29,30,31] to *f*_1_ = 0.02 and *f*_2_ = 0.016, respectively. Generally, the contact damping *c* of the bearing in running state is variable, while the damping coefficient λ is near constant [32,33,34] and is suggested to λ=0.02 [35]. To set the contact stiffness, a normal contact stiffness factor FKN is employed in ANSYS to estimate the contact stiffness based on the material properties and the elemental deformations. FKN is suggested to be the maximum value within the interval [0.1, 1] to avoid penetration and keep the contact stress unchanged using static analysis [36]. Based on the static analysis of bearing under *F_r_* =14 kN, the FKN=0.12 can be empirically determined with *f*_1_ = 0.02, *f*_2_ = 0.016, and λ=0.02. The contact parameters of the FEM model are finally listed in Table 3.

In the FEM simulations, the shaft and inner ring are combined as one volume. The displacement constraining of the model are: the axial degree-of-freedom (DOF) of bearing, the rotating DOF of outer ring, and all the DOFs on the outer surface of bearing seat.

However, the loads of bearing are unknown, include the gravity load of shaft *F_g_*, the eccentric load caused by machining error *F_e_*, and the preload of inner ring *F_ro_*, which are the main factors affecting the vibration response of the bearing. Therefore, the three loads are the sensitive parameters to be updated using the cosine similarity *cos*(*θ*). Referring to the rotating load of bearing in the actual experiment, the rotating speed *n =* 1797 rpm is applied to the inner surface of the inner ring. *F_g_* is preliminary set in the range from 100 N to 1000 N. Meanwhile, *F_e_* is applied to the upper surface of inner ring. According to the general coaxiality requirement of a shaft, the maximum eccentric distance of the shaft is limited to 0.01 mm. Then *F_e_* is accordingly updated in the range of 0 to 0.2 MPa. Moreover, *F_i_* is applied on the inner surface of the inner ring to make each ball fully contact with the raceway. According to Reference [37], the radial preload of inner ring *F_ao_* can be calculated:

(4) $$F_{\mathrm{ao}}=1.13\times 10^{-12}D_{w}^{3}d_{m}\left(1-\gamma^{2}\right)\left(1-\gamma \right)\times Zn^{2}\sin\beta \sin\alpha /f_{1}+1.9F_{r}tg\alpha ,$$

(5) $$F_{\mathrm{ro}}=F_{\mathrm{ao}}\cdot \cos\alpha /S_{i},$$

where *D_w_* denote the diameter of ball; *d_m_* is the inner diameter of bearing; *Z* is the number of balls; *α* = 0.19 rad is the contact angular of the bearing; *β* is the self-rotation angle of ball, *β*=*α*; *γ* denotes the structural parameter of bearing, $\gamma =2D_{w}\cos\alpha /\left(D+d\right)$; *D* and *d* are the outer and inner diameters of bearing (mm), respectively; and *S_i_* is the area of inner surface of inner ring. Using Equation (5), *F_ro_* can be determined.

**Figure 3:**
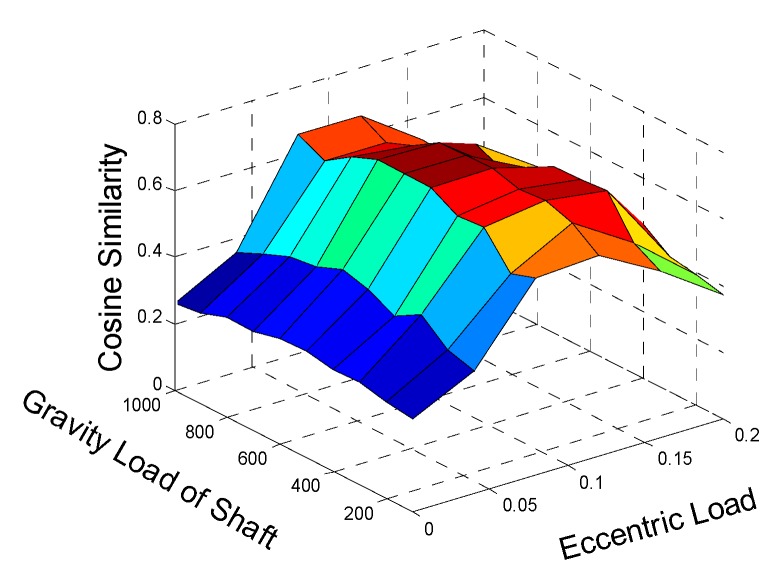
The cosine similarity between measured and simulated signals in normal state.

**Figure 4:**
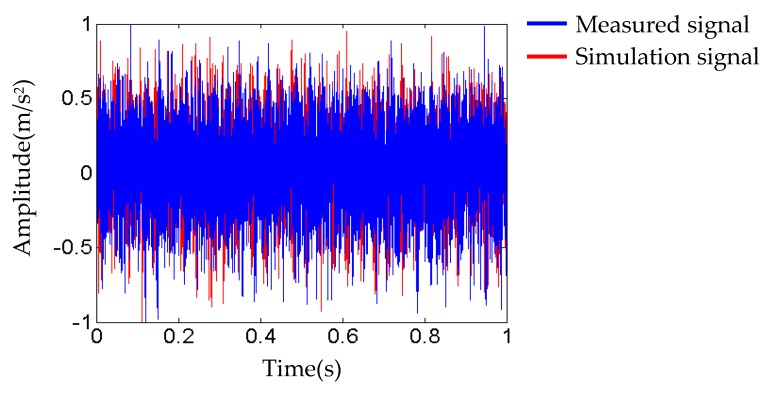
The comparison between measured and simulated signals in normal state.

Through iteratively adjusting the selected load parameters (*F_g_*, *F_e_*, and *F_ro_*) according to the flowchart shown in Figure 1, we finally obtain the maximum cosine similarity *cos*(*θ*) = 0.618. The changing trend of cosine similarity values are shown in Table 4 and Figure 3. Figure 4 shows the comparison between measured and simulated signals when *cos*(*θ*) = 0.618. The two signals are matched well at a certain degree, which proves that the updated FEM model constructed using the relative optimal parameters (shown in Table 3) is agreeable.

### 3.2. Generation of Simulation Fault Training Samples

**Figure 5:**
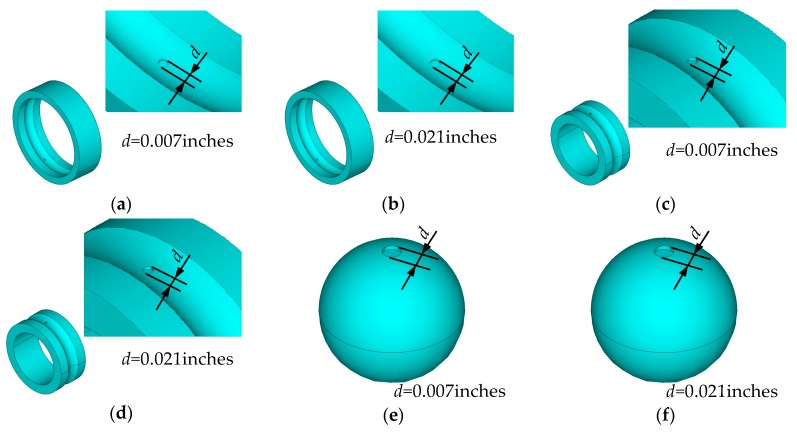
The geometry of faulty bearing: (**a**) Fault type T_1_; (**b**) Fault type T_2_; (**c**) Fault type T_3_; (**d**) Fault type T_4_; (**e**) Fault type T_5_; and (**f**) Fault type T_6._

**Figure 6:**
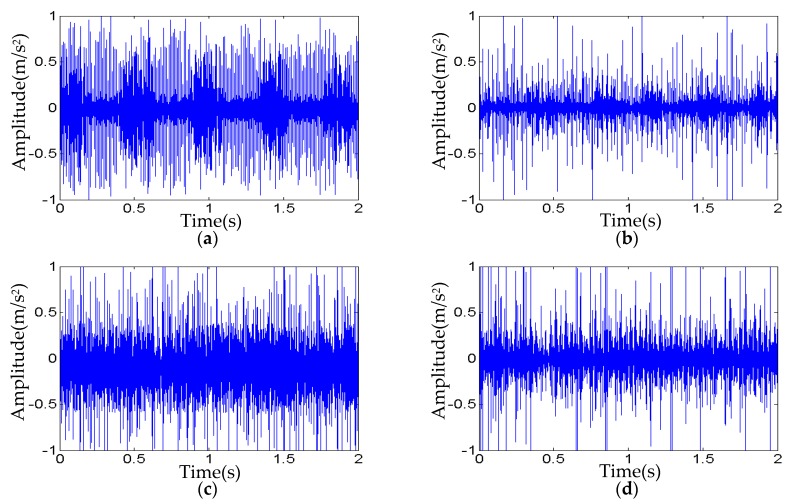

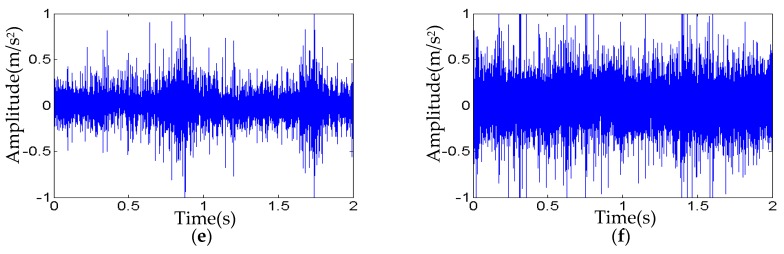
The simulation signals with six types of faults: (**a**) Fault type T_1_; (**b**) Fault type T_2_; (**c**) Fault type T_3_; (**d**) Fault type T_4_; (**e**) Fault type T_5_; and (**f**) Fault type T_6_.

According to bearing data from CWRU, six types of bearing faults (denoted by T_1_, T_2_, T_3_, T_4_, T_5_, and T_6_) are considered, as shown in Figure 5. Using the updated FEM model parameters (shown in Table 4), the FEM models with the above six types of faults are constructed to generate the simulation signals, and the length of each simulation signal is 12,000 data points, as shown in Figure 6. 

For getting satisfactory performance in fault classification, each simulation signal is normalized and divided into 40 sub-signals in time-domain (12,000 data points in total, 300 data points as the length), then calculated the 16 time-domain indexes of each sub-signal. Therefore, for each fault, 16×40=640 indexes are forming a feature vector. Finally, the data as training samples of the six types of faults are a 640×6 matrix.

### 3.3. Experimental Investigations Using a Public Bearing Dataset Based on SVM

In this section, the measured signals corresponding to six types of faults in associates with the simulations are selected from the same Bearing Data Centre at CWRU. 

**Figure 7:**
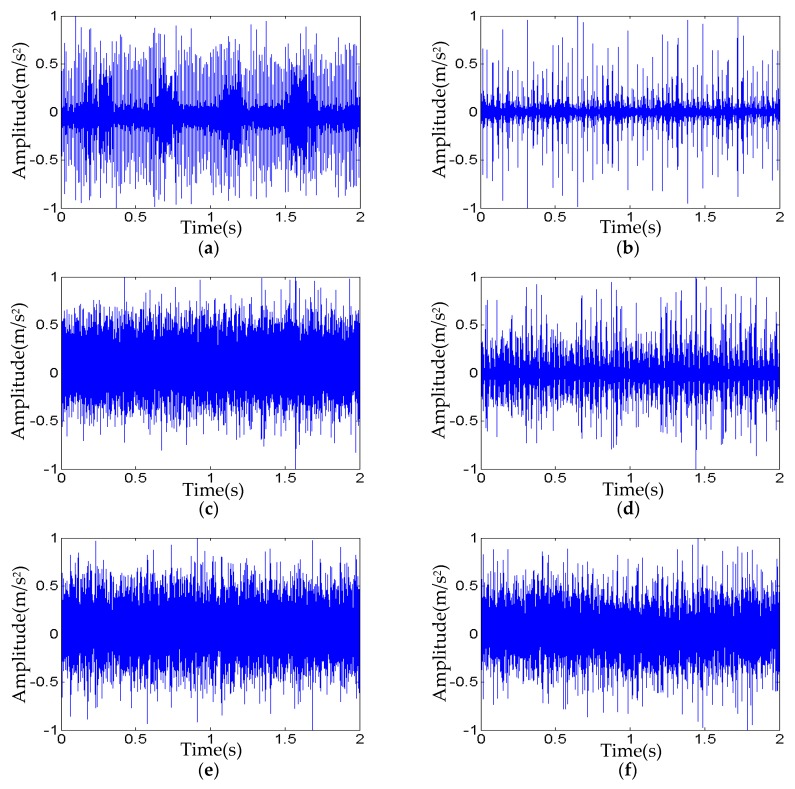
The measured signals with six types of faults: (**a**) Fault type T_1_ (**b**) Fault type T_2_, (**c**) Fault type T_3_, (**d**) Fault type T_4_, (**e**) Fault type T_5_, and (**f**) Fault type T_6._

The six types of bearing faults of the measured signals include the IRF, ORF, and BF with three levels of fault diameters 0.007 inches and 0.021 inches, respectively. Just as the simulation signals, the length of each measured signal is 12,000 data points, which are used for testing SVM. The measured fault signals for testing samples are shown in Figure 7. 

Similar to the FEM simulations for generating testing samples of six faults, each corresponding measured signal is normalized and divided into 40 sub-signals with 300 data points as length in the time domain, and the 16 corresponding time-domain indexes of each sub-signal are calculated. In this fault classification, all six types of fault training samples are all missed. The simulation signals are used to provide the faulty training samples, and the testing samples are all from the measured signals. 

To distinguish the six faults numerically, they are labeled from 1 to 6, respectively. In the process of classification, the adjustable parameters of SVM are selected as *ρ*_1_ = 1.05 and *C* = 10 [27]. Based on the SVM toolkit trained by Franc et al. [38], the six types of faults in bearing are classified and the results are listed in Table 5. It shows that the fault classification accuracy ratios of outer ring, inner ring, and ball using the personalized diagnosis method are 90% (T_1_) and 92.5% (T_2_), 87.5% (T_3_) and 87.5% (T_4_), and 85%(T_5_) and 82.5% (T_6_), respectively. We can see that the classification accuracy ratios are not agreeable. To make a fair comparison, the classification accuracy using the measured signals alone (the training and testing samples are selected from the same measured signals) is also given using the same SVM with parameters *ρ*_1_ = 1.05 and *C* = 10. From Table 5, the relative errors of the present personalized diagnosis method with the measured signals alone are varying from 2.2 % to 12.8%. It notes that the relative errors of inner ring faults T_3_, T_4_, and ball fault T_5_ are a little bit large. The possible reason is the large measured noise of experimental test rigs of Bearing Data Centre at CWRU using the accelerometer mounted on the outer surface of bearing house far away from inner race and balls. In conclusion, the comparison investigations testify that the proposed personalized fault diagnosis method is feasible for judge the fault types of bearings.

Further, the possible way to improve the performance of the personalized diagnosis method is to do in-depth research on the construction of the simpler FEM models, high-performance FEM model updating, and updating more model parameters, and using transfer learning, etc.

## 4. Conclusions

For the application of machine learning methods in intelligent diagnosis of a mechanical system, sufficient fault training samples are the most basic and critical requirement. Based on the advantages of FEM simulation, the problem of lacking samples is solved by using FEM simulation, and the idea of personalized diagnosis based on FEM simulation driving machine learning is put forward. Specifically, a personalized fault diagnosis method based on FEM simulation driving SVM is proposed. The method is applied to diagnosis of the faults in a bearing, and the fault type are distinguished. In the experimental investigations using simulation signals to make up for the lack of faulty training samples, the classification accuracy of three faults located in outer ring, inner ring, and ball are 90% and 92.5%, 87.5% and 87.5%, and 85% and 82.5%, respectively. Finally, the classification results show that the present personalized fault diagnosis method is effective in identifying the faults in bearings. The proposed personalized fault diagnosis method based on FEM simulation driving machine learning can solve many problems, such as providing complete fault samples for various intelligent diagnosis, ensuring that the fault signal is pure without noise interference, etc.

Furthermore, the proposed method is worthy to be widely applied in complex mechanical systems for accurate fault detection.

## Figures and Tables

**Table 1:** Sixteen indexes in time domain.

Index	Equation	Index	Equation
Mean $x_{m}$	$x_{m}=\sum_{i=1}^{N}x_{i}/N$	Average amplitude $x_{ava}$	$x_{ava}=\sum_{i=1}^{N}\left|x\left(i\right)\right|/N$
Standard deviation $x_{std}$	$x_{std}=\sqrt{\sum_{i=1}^{N}{\left(x\left(i\right)-x_{m}\right)}^{2}/N}$	Square root amplitude $x_{sra}$	$x_{sra}={\left(\sum_{i=1}^{N}\sqrt{\left|x\left(i\right)\right|}/N\right)}^{2}$
Variance $x_{var}$	$x_{var}=\sum_{i=1}^{N}{\left(x_{i}-x_{m}\right)}^{2}/N$	Skewness $x_{ske}$	$x_{ske}=\sum_{i=1}^{N}{\left(x\left(i\right)-x_{m}\right)}^{3}/N$
Peak $x_{P}$	$x_{P}=max\left|x\left(i\right)\right|$	Kurtosis $x_{kur}$	$x_{kur}=\sum_{i=1}^{N}{\left(x\left(i\right)-x_{m}\right)}^{4}/Nx_{std}^{4}$
Maximum $x_{max}$	$x_{max}=max\left(x\left(i\right)\right)$	Shape factor SF	$SF=x_{rms}/\left(\sum_{i=1}^{N}\left|x\left(i\right)\right|/N\right)$
Minimum $x_{min}$	$x_{min}=min\left(x\left(i\right)\right)$	Impulse factor IF	$IF=x_{P}/\left(\sum_{i=1}^{N}\left|x\left(i\right)\right|/N\right)$
Peak to peak $x_{pp}$	$x_{pp}=max\left(x\left(i\right)\right)-min\left(x\left(i\right)\right)$	Peak factor PF	$PF=x_{P}/x_{rms}$
Root mean square $x_{rms}$	$x_{rms}=\sqrt{\sum_{i=1}^{N}{\left(x_{i}\right)}^{2}/N}$	Clearance indicator CI	$CI=x_{max}/{\left(\sum_{i=1}^{N}\sqrt{\left|x\left(i\right)\right|}/N\right)}^{2}$

*x* is the data; *N* is the number of data points.

**Table 2:** The element size of bearing.

Component	Element Size (mm)
Outer ring	1
Inner ring	1
Ball	1
Cage	0.5
Shaft	2
Bearing seat	2

**Table 3:** Contact parameters and loading parameters for the FEM model.

Contact Parameters	Value	Loading Parameters	Value
FKN	0.12	*F_g_*	500 N
*f*_1_	0.02	*F_e_*	0.12 MPa
*f*_2_	0.016	*n*	1797 rpm
λ	0.02	*F_ro_*	0.5 MPa

**Table 4:** The experimental design and cosine similarity value of model parameter updating.

cos(θ).		*F_e_* (MPa)					
		0	0.04	0.08	0.12	0.16	0.2
*F_g_* (*N*)	100	0.202	0.268	0.394	0.505	0.403	0.374
	200	0.193	0.292	0.463	0.559	0.446	0.351
	300	0.151	0.255	0.412	0.498	0.501	0.306
	400	0.206	0.311	0.455	0.581	0.455	0.364
	500	0.223	0.351	0.501	0.618	0.451	0.411
	600	0.169	0.271	0.504	0.601	0.507	0.403
	700	0.201	0.336	0.503	0.499	0.473	0.418
	800	0.203	0.227	0.472	0.549	0.437	0.372
	900	0.174	0.294	0.418	0.517	0.422	0.393
	1000	0.161	0.258	0.358	0.458	0.428	0.358

**Table 5:** The classification results using the proposed method (16 indexes) and measured fault signals alone.

Fault Type	Training Samples	Testing Samples	Faults Labels	Classification Accuracy Using the Present Method (%)	Classification Accuracy Using the Measured Signals Alone (%)	Relative Error (%)
T_1_	40	40	1	90%	92%	2.2
T_2_	40	40	2	92.5%	95%	2.6
T_3_	40	40	3	87.5%	95%	7.9
T_4_	40	40	4	87.5%	90%	7.9
T_5_	40	40	5	85%	97.5%	12.8
T_6_	40	40	6	82.5%	87.5%	5.7
